# What are the implications of problem-solving capacity at Primary Health Care in older adult health?

**DOI:** 10.31744/einstein_journal/2022GS6791

**Published:** 2022-06-21

**Authors:** Carolina Aguiar Sant’Anna Siqueri, Gabriel Apolinário Pereira, Giuliana Tamie Sumida, Ana Carolina Cintra Nunes Mafra, Daiana Bonfim, Letícia Yamawaka de Almeida, Camila Nascimento Monteiro

**Affiliations:** 1 Faculdade Israelita de Ciências da Saúde Albert Einstein Hospital Israelita Albert Einstein São Paulo SP Brazil Faculdade Israelita de Ciências da Saúde Albert Einstein, Hospital Israelita Albert Einstein, São Paulo, SP, Brazil.; 2 Hospital Israelita Albert Einstein São Paulo SP Brazil Hospital Israelita Albert Einstein, São Paulo, SP, Brazil.

**Keywords:** Primary Health Care, Health of the elderly, Aging, Family practice, Patient satisfaction, Quality of health care, Health care quality, access, and evaluation, Health services accessibility, Health services for the aged

## Abstract

**Objective:**

To evaluate Primary Health Care attributes and analyze the association between the fulfilment of these attributes and problem-solving capacity of services for elderly patients.

**Methods:**

A cross-sectional, observational, quantitative study. The Primary Care Assessment Tool, designed to assess Primary Health Care attributes, was employed to evaluate elderly users of Primary Care Units located in the south region of the city of São Paulo (SP).

**Results:**

Many attributes assessed at the reference services were considered as unsatisfactory by users. Overall scores were also below the cut-off point. “First contact access – use”, “longitudinality” and “coordination – information system” were the only attributes considered as satisfactory. Also, more than half (62.7%) of respondent patients reported having been referred to specialized services. A combined analysis of these three outcomes revealed users referred to other services had a significantly better perception of Primary Health Care attributes.

**Conclusion:**

The study provides important insights on satisfaction of elderly individuals and the problem-solving capacity of health care services, especially for the study population. Findings reported emphasize the association between Primary Health Care attributes and the problem-solving capacity of health care services at this level.

## INTRODUCTION

Population aging in the last few decades has greatly impacted several sectors of the Brazilian society, including health. To cope with the needs and demands of this growing population, the health care sector had to be adapted and reorganized.^(1)^ Primary Health Care (PHC) plays a key role in this process, not only through the control of risk factors and management of chronic diseases, but also through the coordination of referrals to other services in the health care network.^(2)^

Primary Health Care operates based on four core attributes. Measurable structural and procedural elements pertaining to these attributes are: first contact care, longitudinality, integrality and coordination, and three derivatives, namely family counseling (or centrality), community orientation and cultural competence.^(3)^

Fulfilment of these attributes, associated to service outcomes, can be combined to determine the performance of this sector of the health system.^(3)^ The Primary Care Assessment Tool (PCATool), designed and validated by the Ministry of Health, measures the presence and scope of these attributes, according to experiences and perception of users and health care professionals.^(4)^

Other variables can also be used to examine the effectiveness and determine the performance of PHC, such as hospitalization rates due to conditions that can be resolved at the primary care level and mortality coefficients.^(5,6)^ A more pragmatic approach is the examination of the fundamental premises of PHC: resolution of 85% of health complaints and coordination of referrals and counter-referrals of users in the system.^(7)^The rate of referral to other levels of care can therefore be used to estimate the problem-solving capacity of the first level of care.

Increasing prevalence of multimorbidity, primarily due to chronic conditions, in the elderly population worldwide implies higher demands for specialized care and poses a major challenge to health systems.^(8-10)^

Studies have shown that integrality and accessibility are the most significant attributes for prevention actions and attention to vulnerabilities in elderly populations.^(11,12)^ Hence the need of a network of care.^(13)^ Therefore, problem-solving capacity assessment according to referrals may also provide insights into network integration.

Another important attribute, particularly for elderly individuals with chronic conditions, is integrality.^(14)^ The analysis of care provision in senior health settings must be based on the impact of these factors on the problem-solving capacity of health services, presuming the higher the PCATool score, the greater the problem-solving capacity of a given service.

Notwithstanding the great epidemiologic relevance of this population and respective health conditions in Brazil, little is known about the impacts of PHC attributes and their due implementation in particular settings on the final effectiveness of care.

## OBJECTIVE

To evaluate Primary Health Care attributes and analyze the association between the fulfilment of these attributes and problem-solving capacity of services for elderly patients.

## METHODS

### Type of study

A cross-sectional, observational, quantitative study.

### Sample

The sample comprised users of five PHC (UBS - *Unidade Básica de Saúde*), located in the region of *Supervisão Técnica de Saúde do Campo Limpo* (STSCL), Campo Limpo and Vila Andrade administrative districts, in the south of São Paulo (SP). These units comprised 28 Family Health teams (ESF - *Estratégia Saúde da Família*).

Teams were analyzed in a single model for sample calculation. Therefore, sample size was calculated first, then estimated per team. Individual attribute scores as well as PHC overall and essential scores were analyzed. To obtain a representative sample of each team, a sample size (n) of 12 users per team was calculated (n=336). This sample comprised 83 elderly individuals (aged over 60 years).

### Data collection

Data were collected between July 2019 and January 2020. Users who were at their UBS of origin, whether waiting for consultation or as companions, were interviewed.

Following technical capacitation and training, the PCATool was administered by Medical and Nursing undergraduate students unrelated to the service. No specific sampling strategies were used. Participation was voluntary. Participants were individually approached and signed an informed consent form.

The global scenario brought about by the pandemics introduced several constraints in data collection. Had data been collected as planned (*i.e.*, throughout 2020), a larger sample size would have been achieved.

### Primary Health Care attributes: the data collection instrument Primary Care Assessment Tool-Brasil

The PCATool-Brasil (version for adults) was used. This instrument comprises the following variables: degree of affiliation to the health care service; first contact access - use; first contact access - accessibility; longitudinality; coordination – integration of care; coordination - information system (F),^(4)^ comprising three items (F1, F2 and F3); integrality - services available (G); integrality - services provided (H); family counseling (I) and community orientation (J).^(4)^

Perception of PHC attributes was measured using the PCATool and by specific and overall scores. Scores were presumed to reflect user evaluation of PHC; the higher the score, the better the evaluation. User evaluation of PHC attributes was used as a proxy for satisfaction: the better the evaluation, the higher the level of satisfaction with the service. Total score analysis was based on two values: (essential score) summed mean scores of essential attributes plus degree of affiliation, divided by the number of components and (overall score) summed mean scores of essential attribute and derivative components plus degree of affiliation, divided by the number of components.^(4)^

Attributes scored higher than the cut-off (>6.6 points) were perceived as satisfactory.^(15)^

### Problem-solving capacity

Problem-solving capacity assessment was based on referral rates. In this analysis, referrals were defined as those reported by elderly individuals according to the following question: “In the last year, which professional of (the unit) referred you to a specialist?”.

### Data analysis

Categorical variables were analyzed using descriptive statistics (absolute and relative frequencies). Means and standard deviations of continuous variables were also provided.

Sample size of 83 elderly individuals (54 referred to specialists and 28 not referred) is sufficient to detect differences between groups with medium effect size (h=0.65 and d=0.66 for comparison of proportions and means, respectively) with a level of significance of 5% and statistical power of 80%.

Associations between the medians of PHC attribute scores and referrals per individual were investigated using the Wilcoxon-Mann-Whitney test. Associations between morbidity and problem-solving capacity of PHC were investigated using the χ^2^ test. Analyses were carried out using the R software. The level of significance was set at 5%.

This study is part of the project *Regulação em Saúde: Fatores Relacionados à Resolutividade na Atenção Básica* [Regulation on Health: Factors Related to Problem-solving Capacity in Primary Health Care], approved by the Research Ethics Committees of *Hospital Israelita Albert Einstein* (HIAE), # 3.935.181, CAAE: 06807019.2.0000.0071, and Municipal Health Department of São Paulo, # 3.263.871, CAAE: 06807019.2.3001.0086.

## RESULTS

A total of 83 elderly individuals were interviewed. Of these, 69.9% were female. Mean age of the study population was 67.8 years (minimum and maximum age, 62.9 and 70.7 years, respectively). White and brown races were equally represented (42.2% each). As to socioeconomic profile, 54.2% had studied beyond the elementary level, 61.5% were retired, 54.9% received government benefits, and only 9.9% had health insurance (Table 1).

**Table 1:** Characteristics of the study population

Variable	
Sex	
Male	25 (30.1)
Female	58 (69.9)
Race/color	
White	35 (42.2)
Black	12 (14.5)
Brown	35 (42.2)
Yellow	1 (1.2)
Level of education, course taken	
Did not study	4 (4.8)
Preschool	2 (2.4)
Elementary education	45 (54.2)
Secondary education	26 (31.3)
Higher education	6 (7.2)
Employment status	
Unemployed	13 (16.7)
Self-employed	8 (10.3)
Formal employment	4 (5.1)
Informal employment	5 (6.4)
Retired/pensioner	48 (61.5)
Government benefits	
No	37 (45.1)
Yes	45 (54.9)
Health insurance	
No	73 (90.1)
Yes	8 (9.9)
Ability to see	
Major permanent difficulty	10 (12.0)
Some permanent difficulty	28 (33.7)
No difficulty	45 (54.2)
Hearing ability	
Major permanent difficulty	4 (4.8)
Some permanent difficulty	20 (24.1)
No difficulty	59 (71.1)
Ability to walk	
Major permanent difficulty	12 (14.5)
Some permanent difficulty	23 (27.7)
No difficulty	48 (57.8)
Number of morbidities	2.00 (1.00-3.00)
User suffering from one disease	
No	28 (33.7)
Yes	55 (66.3)
User suffering from two diseases	
No	55 (66.3)
Yes	28 (33.7)
User suffering from three diseases	
No	62 (74.7)
Yes	21 (25.3)
User suffering from four or more diseases	
No	82 (98.8)
Yes	1 (1.2)

Results expressed as n (%) or median (interquartile range).

In this sample, 42.5% of participants were regular users of services (*i.e.*, registered users for more than 10 years) and 61.4% reported multimorbidity (median of two per person, 1;3).

**Figure 1:**
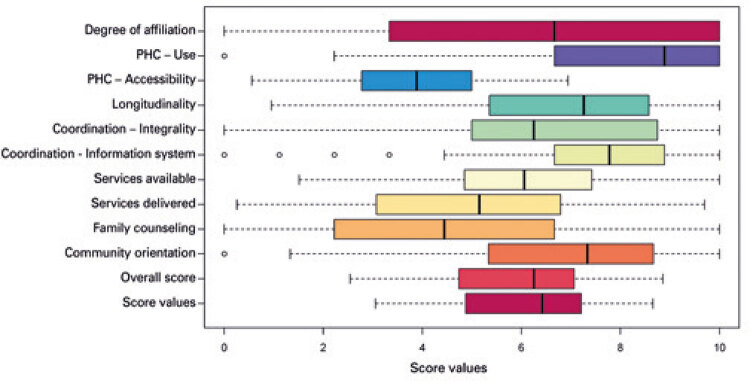
Primary Care Assessment Tool Scores

Primary Care Assessment Tool Scores application (Figure 1) revealed that first contact access - use was the highest rated attribute (median, 8.9). Other attributes perceived as satisfactory were longitudinality and coordination - information system (median of 7.1 and 7.8, respectively).

PHC: Primary Health Care.

First contact access - accessibility and family counseling were the lowest rated attributes (median of 3.6 and 4.4, respectively). Other findings were as follows: coordination – integration of care (6.2), integrality - services available (6.0) and community orientation (6.1).

The degree of affiliation to the health care service (longitudinality attribute), was scored 6.7. Median essential and overall scores in this study were 6.2 (4.7-7.2) and 6.1 (4.6-7.0), respectively.

In this sample, 67.9% of elderly individuals reported not having difficulties to schedule a medical visit at their respective units as needed. As to integrality, 82% of respondents reported their prescriptions were reviewed and discussed by health care professionals. Out of 80 responses, 85% indicated health care professionals were aware of medications taken by patients, and 54.9% revealed lack of advice about fall prevention. With regard to longitudinality, 71.2% of respondents reported professionals were informed of their complete medical history.

The rate of referral of elderly individuals to specialists was 62.7%, indicating a problem-solving capacity of 37.3% (Table 2). Medical specialists with the largest volumes of referrals were ophthalmology (21.13%), cardiology (14.7%) and orthopedics (14.7%).

**Table 2:** Characteristics of the study population according to referral to specialists

Category	Not referred (n=28)	Referred (n=54)	P (*exact test*)
Age	65.81 (62.69-68.69)	68.65 (63.41-71.45)	0.087
Sex			
Male	8 (28.6)	17 (31.5)	1.000
Female	20 (71.4)	37 (68.5)	
Race/color			
White	12 (42.9)	23 (42.6)	0.966
Black	3 (10.7)	8 (14.8)	
Brown	13 (46.4)	22 (40.7)	
Yellow	0	1 (1.9)	
Level of education			
Did not study	3 (10.7)	2 (3.7)	0.522
Preschool	0	2 (3.7)	
Elementary education	17 (60.7)	28 (51.9)	
Secondary education	7 (25.0)	17 (31.5)	
Higher education	1 (3.6)	5 (9.3)	
Employment			
Unemployed	5 (17.9)	9 (8.4)	0.760
Self-employed	2 (7.1)	5 (10.2)	
Formal employment	2 (7.1)	2 (4.1)	
Informal employment	3 (10.7)	2 (4.1)	
Retired/pensioner	16 (57.1)	31 (63.3)	
Benefit			
No	11 (39.3)	26 (49.1)	0.484
Yes	17 (60.7)	27 (50.9)	
Health insurance			
No	23 (85.2)	49 (92.5)	0.432
Yes	4 (14.8)	4 (7.5)	
Ability to see			
Major permanent difficulty	3 (10.7)	7 (13.0)	0.946
Some permanent difficulty	9 (32.1)	19 (35.2)	
No difficulty	16 (57.1)	28 (51.9)	
Hearing ability			
Major permanent difficulty	1 (3.6)	3 (5.6)	1.000
Some permanent difficulty	7 (25.0)	13 (24.1)	
No difficulty	20 (71.4)	38 (70.4)	
Ability to walk			
Major permanent difficulty	3 (10.7)	9 (16.7)	0.250
Some permanent difficulty	11 (39.3)	12 (22.2)	
No difficulty	14 (50.0)	33 (61.1)	
User suffering from one disease			
No	8 (28.6)	20 (37.0)	0.474
Yes	20 (71.4)	34 (63.0)	
User suffering from two diseases			
No	16 (57.1)	39 (72.2)	0.217
Yes	12 (42.9)	15 (27.8)	
User suffering from three diseases			
No	23 (82.1)	38 (70.4)	0.295
Yes	5 (17.9)	16 (29.6)	
Morbidities (median)	2.00 (1.00-3.00)	2.00 (1.00-3.00)	0.916
Overall score (median)	5.35 (4.36-6.53)	6.30 (4.78-7.10)	0.043
Essential score (median)	5.56 (4.31-6.39)	6.58 (5.21-7.22)	0.024

Results expressed as median (interquartile range) or n (%).

**Figure 2:**
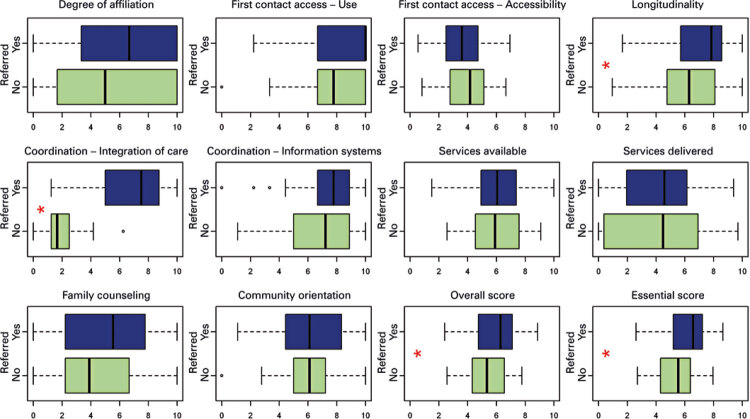
Primary Care Assessment Tool scores according to referral

The following scores differed significantly (p<0.05) between referred and not referred groups: first contact access – use, coordination – care integration, longitudinality, essential and overall scores. Scores were lower among not referred patients (Figure 2).

## DISCUSSION

Data analysis revealed that many attributes assessed at services of origin were perceived as unsatisfactory by service users. Overall scores were also below the cut-off. First contact – access, longitudinality and coordination – information system were the only attributes perceived as satisfactory. The fact that more than half (62.7%) of respondents reported having been referred to a specialty service shows that the perception of PHC attributes as satisfactory was significantly greater among referred users.

This study addressed an elderly population of health system users. These individuals are primarily PHC users and possibly rely on these services for medical care.^(16)^ Also, given the substantial prevalence of multimorbidity in this age range and the significance of ongoing medical care for positive health outcomes,^(17)^ longitudinality is a major concern. Finally, appropriate health data recording and use ensures easy and seamless exchange of information^(18)^ between the different levels of the health care system. However, since PHC is an integrated system that relies on different attributes for appropriate functioning,^(2)^ these attributes must be fulfilled to ensure health care quality.

First contact access – accessibility, and community orientation (derivative attribute) were the lowest rated attributes in this study. First contact access - accessibility scores suggest structural factors inherent to the health system may act as barriers to care, implying undisputable losses to the individuals and services expenses.^(15)^

The impact of community orientation tends to be less significant, given the derivative nature of this attribute.^(2)^ However, this property allows comprehensive assessment of health care deficiencies and flaws in a given region or collectivity^(19)^ and is vital for appropriate health care provision.

To achieve a more satisfactory fulfillment of these attributes, the requirements of different populations may need to be individualized^(19)^ and medical care tailored to community settings (*i.e.*, local availability of resources for health promotion) or access constraints (*i.e.*, identification and removal of physical barriers, *e.g.*,).

Prates et al.^(15)^ carried out a systematic review of publications addressing the assessment of attributes in different contexts and countries, and by different methods. Highest rating attributes in that review are consistent with findings of this study (first contact access – use (71.4%), longitudinality (64.0%) and coordination – care integration (54.5%).

In a different study with elderly individuals,^(11)^ longitudinality was the highest rated attribute (mean score, 7.3; PCAT tool). Integrality was the second lowest rated (mean score, 4.7; PCAT tool). Likewise, integrality - services available, was the third lowest rated attribute in this study. Consistent results emphasize the robustness of the PCAT tool and support the reliability of assessments.^(15,20)^ User dissatisfaction due to poor accessibility, care coordination and integrality, among other factors, has been emphasized in prior studies.^(21)^

More than 80% of respondents reported that medical professionals are informed of and discuss about their prescriptions. This is an extremely relevant factor in the relation between user satisfaction and problem-solving capacity of health services for the study population. The issue of polypharmacy, and the vulnerability of elderly individuals to polypharmacy in particular,^(22)^ highlight the implications of prescription revision for positive outcomes and health care quality in this population. Growing frailty and increased risk of falls in elderly individuals emphasize the significance of patient education in this regard.^(23)^ The fact that a substantial number of respondents reported not having received guidance about fall prevention indicates an opportunity for improvement in the integrality attribute.

Analysis based on referral rates revealed a problem-solving capacity of 37.3%. This study involved elderly individuals with chronic diseases and multimorbidity, most of them suffering from more than two comorbidities, which implies more referrals. Of elderly individuals analyzed in National Health Survey (PNS - *Pesquisa Nacional de Saúde )* those with more morbidities were also the ones who used health care services more often and attributed higher scores (6.1 in a 0-10 scale) to these services.^(24)^

Combined analysis of attributes and problem-solving capacity revealed that patients referred to other services tended to rate their UBS of origin higher. Importantly, health professionals working at these units act as system gatekeepers.^(25)^ Ideally, referrals should be a matter of continuity rather than replacement of PHC, to achieve the desired integrality.^(26)^ However, referral increases the chances of meeting user needs of specialized, high complexity care.

Of notice, according to Costa et al.^(26)^ services that fulfill their PHC attributes should be able to provide better care to users, with less need of referral and not otherwise, as shown in this study. Greater attention on the part of managers and team members is needed to offer more standardized care, with resultant benefits to users.

The cultural perspective of health care service use must also be accounted for. Specialty referral is the primary goal of many UBS users, whereas the primary level of care and its inherent problem-solving capacity are not duly appreciated.^(27)^ Therefore, aside from structural changes, user expectations must be understood and respected for PHC consolidation.

The construction of a more robust PHC, with greater problem-solving capacity for this population, and aimed to deliver better health outcomes in chronic conditions, can be achieved through a more effective articulation of services in health care networks ( RAS - *Redes de Atenção à Saúde*).^(2)^ The identification and resolution of well-established lack of family and community medicine competencies required to detect and stratify risk factors, such as frailty,^(28,29)^ can help to optimize care protocols for this population and prevent negative impacts and unfavorable outcomes.

This study has limitations, such as small sample size due to challenges in data collection.

Implications of results reported range from the need to improve the health care system and the PHC level to greater attention to users and their perception of health care. Finally, the fact that problem-solving capacity can be assessed in several ways and that referral rates may contain inconsistencies, must be emphasized.

## CONCLUSION

The study provides important insights into the perception of elderly individuals and the problem-solving capacity of health care services, and is of relevance to the study population. Findings reported emphasize the association between Primary Health Care attributes and the problem-solving capacity of health care services at this level, and highlights areas for improvement (frailty, access, and network articulation).

## References

[1] Miranda GM, Mendes AC, Silva AL. O envelhecimento populacional brasileiro: desafios e consequências sociais atuais e futuras. Rev Bras Geriatr Gerontol. 2016;19(3):507-19.

[2] Brasil. Ministério da Saúde. Secretaria de Atenção à Saúde. Departamento de Atenção Especializada e Temática (DAET). Coordenação Saúde da Pessoa Idosa (COSAPI). Diretrizes para o cuidado das pessoas idosas no SUS: proposta de modelo de Atenção Integral. XXX Congresso Nacional de Secretarias Municipais de Saúde. Brasília (DF): Ministério da Saúde; 2014 [citado 2021 Jun 23]. Disponível em: https://bvsms.saude.gov.br/bvs/publicacoes/diretrizes_cuidado_pessoa_idosa_sus.pdf .

[3] Brasil. Ministério da Saúde. Secretaria de Políticas de Saúde. Departamento de Atenção Básica. Organização das Nações Unidas, para a Educação, a Ciência e a Cultura (UNESCO). Atenção Primária: equilíbrio entre necessidades de saúde, serviços e tecnologia. Brasília (DF): UNESCO, Ministério da Saúde; 2002. p. 726 [citado 2021 Jun 23]. Disponível em: https://www.nescon.medicina.ufmg.br/biblioteca/imagem/0253.pdf

[4] Brasil. Ministério da Saúde. Secretaria de Atenção à Saúde. Departamento de Atenção Básica. Manual do instrumento de avaliação da atenção primária à saúde. Primary Care Assessment Tool PCATool-Brasil. Brasília (DF): Ministério da Saúde; 2010 [citado 2021 Jun 23]. [Série A. Norma e Manuais Técnicos]. Disponível em: https://bvsms.saude.gov.br/bvs/publicacoes/manual_avaliacao_ atencao_primaria.pdf

[5] Nedel FB, Facchini LA, Bastos JL, Martín-Mateo M. Conceptual and methodological aspects in the study of hospitalizations for ambulatory care sensitive conditions. Cien Saude Colet. 2011;16(Suppl1):1145-54. 10.1590/s1413-81232011000700046 21503462

[6] Sala A, Mendes JD. Perfil de indicadores da atenção primária à saúde no estado de São Paulo: retrospectiva de 10 anos. Saúde Soc. 2011;20(4):912-26.

[7] Organização Pan-Americana da Saúde (OPAS). Organização Mundial da Saúde (OMS). Conselho Nacional de Secretários de Saúde (CONASS). O cuidado das condições crônicas na atenção primária à saúde: o imperativo da consolidação da estratégia da saúde da família. Brasília (DF): OPAS, OMS, CONASS; 2012 [citado 2021 Jun 23]. Disponível em: https://bvsms.saude.gov.br/bvs/publicacoes/cuidado_condicoes_atencao_primaria_saude.pdf

[8] Starfield B. Challenges to primary care from co- and multi-morbidity. Prim Health Care Res Dev. 2011;12(1):1-2. 10.1017/S1463423610000484 21426609

[9] Schenker M, Costa DH. Advances and challenges of health care of the elderly population with chronic diseases in Primary Health Care. Cien Saude Colet. 2019;24(4):1369-80. 10.1590/1413-81232018244.01222019 31066839

[10] Reeves D, Pye S, Ashcroft DM, Clegg A, Kontopantelis E, Blakeman T, et al. The challenge of ageing populations and patient frailty: can primary care adapt? BMJ. 2018;362:k3349. 10.1136/bmj.k3349 30154082

[11] Araújo LU, Gama ZA, Nascimento FL, Oliveira HF, Júnior Azevedo HJ. Avaliação da qualidade da atenção primária à saúde sob a perspectiva do idoso. Cien Saude Colet. 2014;19(8):3521-32. 10.1590/1413-81232014198.21862013 25119091

[12] Augusto DK, Lima-Costa MF, Macinko J, Peixoto SV. Fatores associados à avaliação da qualidade da atenção primária à saúde por idosos residentes na Região Metropolitana de Belo Horizonte, Minas Gerais, 2010. Epidemiol Serv Saúde. 2019;28(1):e2018128. 10.5123/S1679-49742019000100017 30970074

[13] Oliveira MR, Veras RP, Cordeiro HA. A importância da porta de entrada no sistema: o modelo integral de cuidado para o idoso. Physis (Rio J.). 2018;28;(4):e280411.

[14] Silva LB, Silva PA, Santos JF, Silqueira SM, Borges EL, Soares SM. Estratos de risco e qualidade do cuidado à pessoa idosa na Atenção Primária à Saúde. Rev Lat Am Enfermagem. 2019;27:e3166. 10.1590/1518-8345.2968.3166 PMC6781381 31596406

[15] Prates ML, Machado JC, Silva LS, Avelar PS, Prates LL, Mendonça ET, Cotta RM, et al. Performance of primary health care according to PCATool instrument: a systematic review. Cien Saude Colet. 2017;22(6):1881-93. Review. 10.1590/1413-81232017226.14282016 28614508

[16] Macinko J, Andrade FB, Souza Junior PR, Lima-Costa MF. Primary care and healthcare utilization among older Brazilians (ELSI-Brazil). Rev Saude Publica. 2018;52 Suppl 2(Suppl 2):6s. 10.11606/S1518-8787.2018052000595 PMC6254960 30379279

[17] Cunha EM, Giovanella L. Longitudinalidade/continuidade do cuidado: identificando dimensões e variáveis para a avaliação da Atenção Primária no contexto do sistema público de saúde brasileiro. Cien Saude Colet. 2011; 16(Suppl 1):1029-42. 10.1590/s1413-81232011000700036 21503452

[18] Almeida PF, Medina MG, Fausto MC, Giovanella L, Bousquat A, Mendonça MH. Coordenação do cuidado e Atenção Primária à Saúde no Sistema Único de Saúde. Saúde Debate. 2018;42(1):244-60.

[19] Reichert AP, Leônico AB, Toso BR, Santos NC, Vaz EM, Collet N. Family and community orientation in children’s primary healthcare. Cien Saude Colet. 2016;21(1):119-27. 10.1590/1413-81232015211.05682014 26816170

[20] Stein AT. A avaliação dos serviços de saúde deve ser realizada com instrumentos validados. Epidemiol Serv Saúde. 2013;22(1):179-81.

[21] Lima Ede F, Sousa AI, Primo CC, Leite FM, Lima Rde C, Maciel EL. An assessment of primary care atributes from the perspective of female healthcare users. Rev Lat Am Enfermagem. 2015;23(3):553-9. 10.1590/0104-1169.0496.2587 PMC4547080 26155006

[22] Secoli SR. Polifarmácia: interações e reações adversas no uso de medicamentos por idosos. Rev Bras Enferm. 2010;63(1):136-40. 10.1590/s0034-71672010000100023 20339769

[23] Gasparotto LP, Falsarella GR, Coimbra AM. As quedas no cenário da velhice: conceitos básicos e atualidades da pesquisa em saúde. Rev Bras Geriatr Gerontol. 2014;17(1):201-9.

[24] Brasil. Ministério da Economia. Instituto Brasileiro de Geografia e Estatística (IBGE). Agência IBGE Notícias. PNS 2019: Quem mais utiliza o SUS avaliou mais positivamente a qualidade dos serviços de Atenção Primária à Saúde. Rio de Janeiro: IBGE; 2020. Disponível em: https://agenciadenoticias.ibge.gov.br/agencia-sala-de-imprensa/2013-agencia-de-noticias/releases/29203-pns-2019-quem-mais-utiliza-o-sus-avaliou-mais-positivamente-a-qualidade-dos-servicos-de-atencao-primaria-a-saude

[25] Gérvas J, Pérez Fernández M. El fundamento científico de la función de filtro del médico general. Rev Bras Epidemiol. 2005;8(3):205-18.

[26] Costa JP, Jorge MS, Vasconcelos MG, Paula ML, Bezerra IC. Resolubilidade do cuidado na atenção primária: articulação multiprofissional e rede de serviços. Saúde Debate. 2014;38(103):733-43.

[27] Sala A, Luppi CG, Simões O, Marsiglia RG. Integralidade e atenção primária à Saúde: avaliação na perspectiva dos usuários de unidades de saúde do município de São Paulo. Saúde Soc São Paulo. 2011;20(4):948-60.

[28] Mulla E, Orton E, Kendrick D. Is proactive frailty identification a good idea? A qualitative interview study. Br J Gen Pract. 2021;71(709):e604-e13. 10.3399/BJGP.2020.0178 PMC8252857 33657008

[29] Alharbi K, van Marwijk H, Reeves D, Blakeman T. Identification and management of frailty in English primary care: a qualitative study of national policy. BJGP Open. 2020;4(1):bjgpopen20X101019. 10.3399/bjgpopen20X101019 PMC7330193 32184213

